# Supplementary material for: A Subset of Nucleus Accumbens Neurons Receiving Dense and Functional Prelimbic Cortical Input Are Required for Cocaine Seeking
Source: Front Cell Neurosci. 2022 Feb 24;16:844243. doi: 10.3389/fncel.2022.844243 (PMC8907444; doi:10.3389/fncel.2022.844243)
Supplement: Supplementary file 1 [file Data_Sheet_1.pdf]

Supplemental information

Materials and methods

Animal subjects and surgery

Male Sprague-Dawley rats were purchased from Charles Rivers Laboratories (Wilmington, MA, ~275-300 g). Rats were single-housed in a temperature and humidity controlled vivarium with a 12:12 hour reverse light/dark cycle (lights off at 6AM). All experiments were conducted during the dark phase. Animals were allowed to acclimate for 5 days prior to undergoing experiments with food and water available *ad libitum*. Prior to surgery, animals were anesthetized with a ketamine (66 mg/kg, i.p.) and xylazine (1.33 mg/kg) cocktail and received ketorolac (Sigma, 2 mg/kg i.p.) for analgesia. Animals undergoing intravenous (i.v.) cocaine (or yoked-saline) SA were implanted with a chronic indwelling catheter as previously described (12, 30). Catheters exited via a small incision in the shoulder blades. Following surgery, catheters were flushed with Cefazolin to prevent bacterial infection. Catheters were flushed daily for 5 days post-surgery with 50 µl of taurolidine citrate catheter lock solution (Access technologies) to maintain patency. Animals were then secured in a stereotaxic apparatus (David Kopf instruments, model 942). Rats then received an intra-prelimbic (PrL) cortical (AP: + 2.8mm, ML: +/- 0.6mm, DV: -3.8 mm relative to bregma) virus injection and an intra-nucleus accumbens core (NAcore, AP: +1.7mm, ML: +/- 1.6mm, DV: -7mm relative to bregma) virus injection (see table 1 for viral vector information). Injections were performed over a period of 5 minutes using a Nanoject II (0.75 µl/ hemisphere, Drummond Scientific, Broomall, PA). Injectors were left in place for 5 minutes to facilitate diffusion away from the injection site, then slowly retracted. The incision was then sutured closed and animals were allowed to recover from anesthesia.

Perfusions and immunohistochemistry

Immunohistochemistry was performed as previously described (8, 31, 65). Briefly, rats were transcardially perfused with 5% buffered formalin (150 ml) following a pre-flush with 0.1M phosphate buffered saline (PBS, 120 ml) at a rate of 60 ml/minute. For dendritic spine morphology and astrocyte association at dendritic spine experiments, rats were pre-flushed with 0.1M phosphate buffer (PB, 120 ml) followed by a transcardial perfusion with 4% granular paraformaldehyde (180 ml) in 0.1M PB. Brains were extracted and post-fixed in the same fixative for 24 hours. A vibrating microtome (Leica) was used to section brains. Free-floating coronal sections containing the PrL cortex (60 µm) and NAcore (60-80 µm) were blocked in 0.1M PBS with 2% Triton X-100 (PBST) with 2% normal goat serum (NGS, Jackson Immuno Research, Westgrove, PA) for 2 hours at room temperature with agitation. Sections were then incubated overnight at 4ºC with agitation in the appropriate primary antisera diluted in 2% PBST with 2% NGS (see Table 2), washed 3 times for 5 minutes in PBST, then incubated in the appropriate secondary antisera (see Table 2) diluted in PBST with 2% NGS for 2-5 hours at room temperature with agitation. All secondary antisera were raised in goat, conjugated to Alexa fluorophores, were used at a concentration of 1:1000, and were purchased from Invitrogen (Carlsbad, CA). Sections were then washed 3 times for 5 minutes in PBST, mounted on SuperFrost+ slides, and cover slipped with ProLong™ Gold Antifade. Slides were stored at 4ºC protected from light until imaging.

Microscopy

Mapping of virus expression and, colocalization of mCherry/Flag with different cell types, and Fos

All imaging was performed by an investigator blind to experimental conditions. To determine accuracy of microinjections in both the PrL cortex and NAcore, a Leica THUNDER Imager Tissue equipped with 488, Cy3, and Ct5 filter cubes (Leica microsystems) was used. Exposure time was

held relatively constant between images. Virus expression was then mapped by an investigator blind to experimental conditions, and expression profiles were overlaid to generate a histology map.

AAV1 vectors expressing Cre recombinase are known to have transsynaptic properties whereby transduction of neurons, and thus expression of Cre, downstream from the injection site is known to occur in a manner dependent on vesicular release as well as the strength of the connection between the neurons (33, 34). To determine what cell types in the NAcore are transduced by AAV-CamKIIα-Cre when injected into the PrL cortex, tissue from Yoked Saline controls, animals undergoing 15 minutes of reinstatement, and animals undergoing 2 hours of cue-induced reinstatement were immunohistochemically processed for various markers and a Leica SP8 laser-scanning confocal microscope was used to image virally-transduced neurons in the NAcore in addition to either NeuN, pre-pro Enkephalin (ppENK), nNOS, Parvalbumin (PV), or Choline acetyltransferase (ChAT) in separate IHC runs. mCherry/Flag were imaged using an OPSL 552nm laser line whereas all other markers were imaged using a Diode 638nm laser line. Pinhole size, laser power, gain, and voxel size were held constant between images within each experimental IHC run and images were acquired with either a 10X or 20X air objective, but the same objective was used throughout the experimental run for each marker.

For detection of Fos, virally-transduced neurons (mCherry$^+$) and Fos$^+$ nuclei in the NAcore from animals undergoing cue-induced reinstatement were imaged with a 10X objective with 2X digital zoom. Care was taken to ensure that the field imaged contained mCherry$^+$ neurons within each quadrant and that images were only acquired in the NAcore. As before, all parameters were held constant between images.

Imaris analyses

Colocalization of mCherry/Flag with different cell types and Fos activated neurons

All Imaris image analyses were performed on 3D reconstructed Z-stacks by an investigator blind to experimental conditions. Non-deconvolved images were exported to Imaris (v 9.0, Bitplane) for analyses. For all images, a constant baseline subtraction was applied to the non-mCherry/Flag channel in order to apply a threshold cutoff. Next, the spot tool in Imaris was used to semi-manually label $mCherry^+/Flag^+$ cells as well as $NeuN^+$, $nNOS^+$, $ppENK^+$, PV^+, $ChAT^+$, or Fos^+ cells/nuclei. Due to the low population of interneurons, the number of $mCherry^+/interneuron^+$ neurons were manually counted and normalized to the total number of $mCherry^+$ cells or the total number of interneurons in the image to generate a percentage of total $mCherry^+$ cells that were $nNOS^+$, PV^+, or $ChAT^+$ as well as the inverse (percentage of interneurons that were transduced). For ppENK and NeuN, iterative processing and analyses were performed whereby spots were built on $mCherry^+/Flag^+$ cells, then the ppENK or NeuN signal contained within the transduced cells was masked, and spots were built on the new channel as well as the original channel. For Fos, the colocalize spots MatLab extension (5 µm threshold) was used after spots were built on $mCherry^+$ cells and Fos^+ nuclei. Exported variables included the number of $mCherry^+/Flag^+$ cells, the number of co-labeled (of Fos^+) cells, the percentage of $mCherry^+/Flag^+$ cells that were co-labeled, as well as the percentage of $NeuN^+$, $nNOS^+$, $ppENK^+$, PV^+, or $ChAT^+$ cells that were virally-transduced. The number of Fos^+ neurons was normalized to the dataset volume (in μm^3). For Fos analyses, data was collapsed across hemispheres, then sections, and expressed as an animal average.

Supplemental Figure Legends

Figure S1. Z-series of astrocyte association at dendritic spines and intensity control data, pertaining to Figure 4. A) Individual Z-steps showing an individual spine (marked with an arrow) with Lck-GFP expression surrounding the spine at 13 different focal planes, as well as a 3D render of the same spine. Bottom right – Surface rendering of the dendritic spine and astrocyte membrane surrounding the spine with the spine head rendered as a sphere. Grey area indicates the expanded spine head sphere in 300 nm in all directions. Green render indicates astrocyte in association region of interest (ROI). B) Average Lck-GFP intensity in the association ROI for each dendrite segment analyzed for yoked saline and cocaine cue reinstating animals. There was no difference between groups (t(92)=0.482, *p*=0.631).

Figure S2. Behavior data pertaining to Figure 1. A-B) Sucrose SA and extinction (A) and reinstatement (B) lever pressing for animals undergoing electrochemical recordings (*n*=5) during reinstatement. *p<0.05 compared to extinction.

Figure S3. Cue-induced Fos is increased in cocaine and sucrose animals compared to yoked saline control animals. A) Compared to yoked saline controls (*n*=4, from dendritic spine and astrocyte association experiment – Figure 4), cocaine and sucrose animals expressing mCherry control in $\text{NAcore}^{\text{PrL}}$ neurons show increased Fos in the NAcore (One-way ANOVA: F(2,21)=8.126, *p*=0.0024, Dunnett's post hoc, $p<0.05$). B) Fos was also increased in $\text{NAcore}^{\text{PrL}}$ neurons in the two cocaine groups compared to yoked saline (Brown-Forsythe-corrected ANOVA: F(2,18.92)=20.14, $p<0.0001$, Dunnett's T3 multiple comparison, $p<0.01$). C) Representative Fos expression from a yoked saline control animal. Scale bars = 50µm (left) and 20 µm (right).

Figure S4. Estimates of $\text{NAcore}^{\text{PrL}}$ D2-expressing neuron frequency, pertaining to Figure 4. A) ~50% of cells labeled for dendritic spine morphology measurements were ppENK^+. There was no difference between groups in the percent of labeled neurons that were ppENK^+ (t(19)=0.44, *p*=0.663). B) Representative Flag-tagged-Flex-smFP neurons (Red) and immunohistochemical detection of ppENK (green). Arrow heads point to $\text{Flag}^+/\text{ppENK}^+$ neurons. Asterisks indicate $\text{Flag}^+/\text{ppENK}^-$ neurons. Scale bar=50µm. Tissue was processed from animals expressing Flex-smFP undergoing yoked-saline and sacrificed 24 hours after the final extinction session (*n*=3) or 15 minutes into cued reinstatement of cocaine seeking (*n*=3).

Figure S5. There were no changes in $\text{NAcore}^{\text{PrL}}$ dendritic spine morphology or astrocyte association at spine heads 24 hours after the final cocaine SA session. A) Histology map of viral spread in the PrL cortex as revealed by Cre immunohistochemistry. B) Cocaine (*n*=4) and yoked saline (*n*=4) ILP, ALP, and infusions earned over the last 10 days of SA. C-D) There was no difference in spine d_H (C - unpaired t-test, t(49)=0.834, *p*=0.408) or (D – unpaired t-test, t(49)=0.101, *p*=0.92) dendritic spine density on $\text{NAcore}^{\text{PrL}}$ neurons 24 hours after the final SA session. E-G) There was no difference in the percent of spines per dendrite that showed no association (E – unpaired t-test, t(49)=1.392, *p*=0.17) nor the average association at dendritic spine heads (F – Welch's-corrected t-test, t(38.78)=0.647, *p*=0.522), and there was no specific dendritic spine d_H bins that showed any alterations in percent association at spine heads (G, significant spine d_H bin by treatment interaction, (F(5,229)=2.768, *p*=0.02, no significant Bonferroni post-hoc test).

Supplemental Tables

Figure	Test	Comparison	Statistics
1C	Two-way RM ANOVA	hM4Di vs. mCherry, ALPs during SA	Non-significant session by group interaction (F(9,189)=0.971, *p*=0.465)
1C	Two-way RM ANOVA	hM4Di vs. mCherry Infusions earned during SA	Non-significant session by group interaction (F(9,189)=1.320, *p*=0.229)
1E	Two-way RM ANOVA	hM4Di vs. mCherry ALPs across time	Non-significant session by group interaction (F(7,147)=0.798, *p*=0.599)
3E	Two-way RM ANOVA	hM4Di vs. mCherry ALPs during SA	Non-significant session by group interaction (F(9,189)=1.089, *p*=0.372)
3E	Two-way RM ANOVA	hM4Di vs. mCherry Infusions earned during SA	Non-significant session by group interaction (F(9,189)=0.761, *p*=0.65
3E	Mixed-effects model	hM4Di vs. mCherry ALPs during extinction	Non-significant session by group interaction: F(6,101)=0.423, *p*=0.863)
3F	Two-way RM ANOVA	hM4Di vs. mCherry ILPs during extinction and reinstatement	Non-significant group by test interaction (F(1,21)=0.471, *p*=0.5)
3H	Two-way RM ANOVA	hM4Di vs. mCherry ALPs during SA	Non-significant session by group interaction (F(9,126)=1.278, *p*=0.255)
3H	Two-way RM ANOVA	hM4Di vs. mCherry pellets earned during SA	Non-significant session by group interaction: (F(9,126)=1.749, *p*=0.085)
3H	Mixed-effects model	hM4Di vs. mCherry ALPs during extinction	Non-significant session by group interaction (F(5,65)=0.394, *p*=0.851)
3I	Two-way RM ANOVA	hM4Di vs. mCherry ILPs during extinction and reinstatement	Non-significant session by group interaction (F(1,14)=3.452, *p*=0.084)

Table S1. Non-significant behavioral data.

Primary antisera	Host species	Concentration	Source	RRID (AB_)	Immunogen	Secondary antisera
GFP	Chicken	1:2000	Abcam, ab13970	300798	Recombinant full-length protein	anti-chicken 488
cFos	Rabbit	1:1000	Synaptic Systems, 226 003	N/A	Synthetic peptide	anti-rabbit 647
Flag	Mouse	1:1000	Sigma, F1804	262044	Peptide sequence DYKDDDDK	anti-mouse 594
mCherry	Chicken	1:2000	LS Biosciences, LC-C204825	2716246	Recombinant full-length protein	anti-chicken 594
Cre	Mouse	1:1000	Millipore, MAB3120	2085748	Cre recombinase fusion protein	anti-mouse 647
Pre-pro Enkephalin	Rabbit	1:1000	Neuromics, RA14124	2532106	Peptide sequence	anti-rabbit 647
NeuN	Mouse	1:1000	Millipore, MAB377	2298772	Purified cell nuclei	anti-mouse 647
nNOS	Rabbit	1:1000	Millipore, AB5380	91824	Recombinant human nNOS	anti-rabbit 647
Choline acetyltransfer ase	Mouse	1:1000	Millipore, AMAB91130	2665812	Peptide sequence	anti-mouse 647
Parvalbumin	Mouse	1:1000	Millipore, MAB1572	2174013	Parvalbumin purified from frog muscle	anti-mouse 647

Table S2. Key resource table
