# Supplementary figures and images for: A Subset of Nucleus Accumbens Neurons Receiving Dense and Functional Prelimbic Cortical Input Are Required for Cocaine Seeking
Source: Front Cell Neurosci. 2022 Feb 24;16:844243. doi: 10.3389/fncel.2022.844243 (PMC8907444; doi:10.3389/fncel.2022.844243)

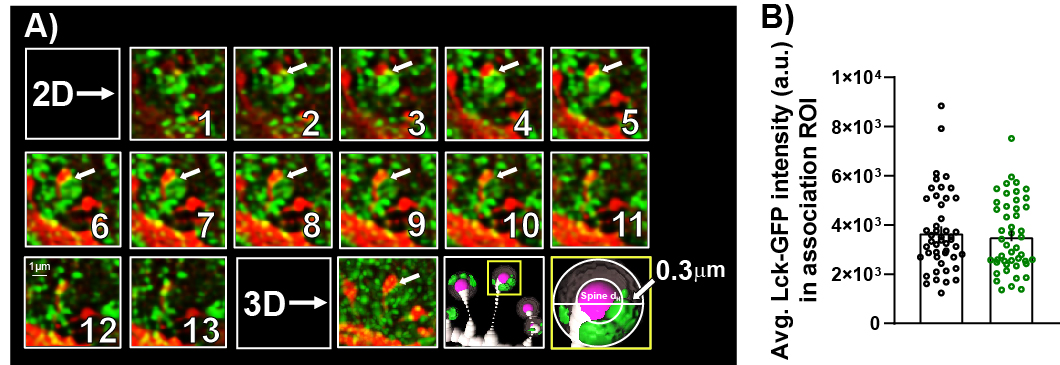

Supplement: Supplementary file 2 [file Image_1.JPEG]

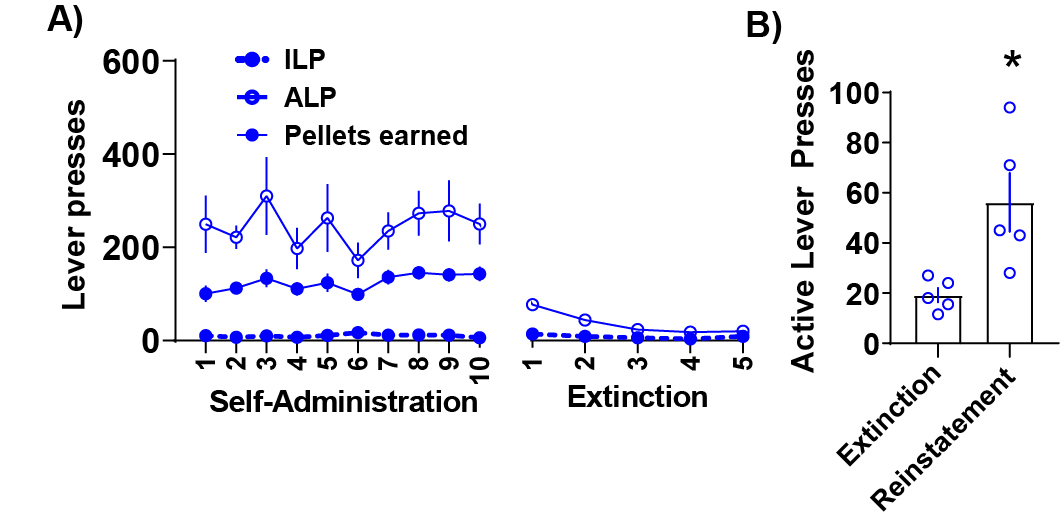

Supplement: Supplementary file 3 [file Image_2.JPEG]

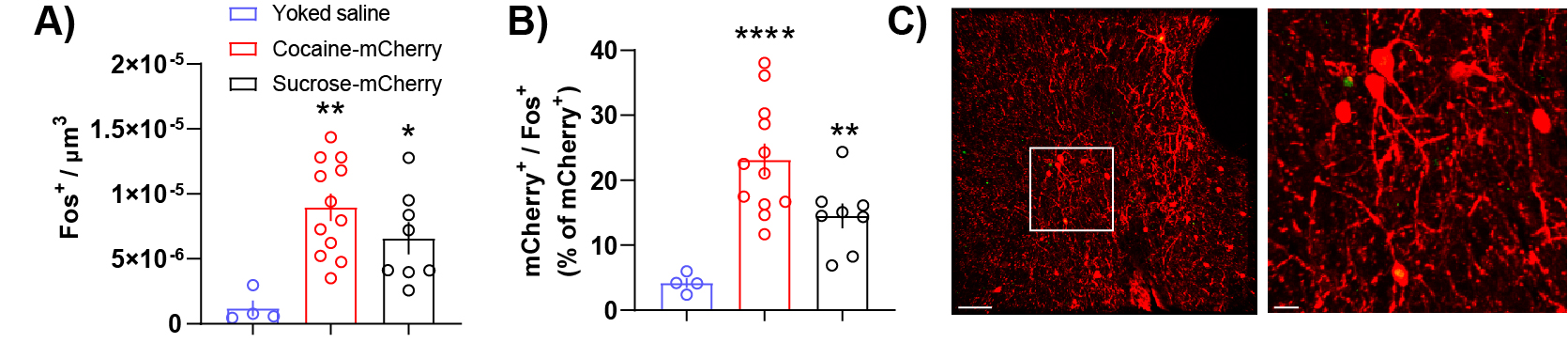

Supplement: Supplementary file 4 [file Image_3.JPEG]

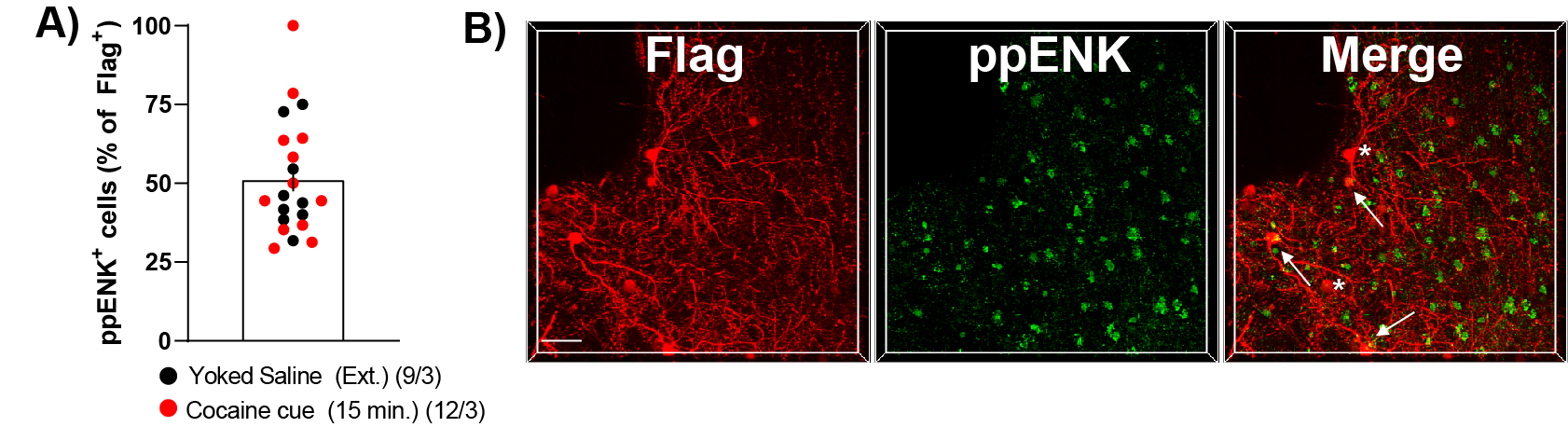

Supplement: Supplementary file 5 [file Image_4.JPEG]

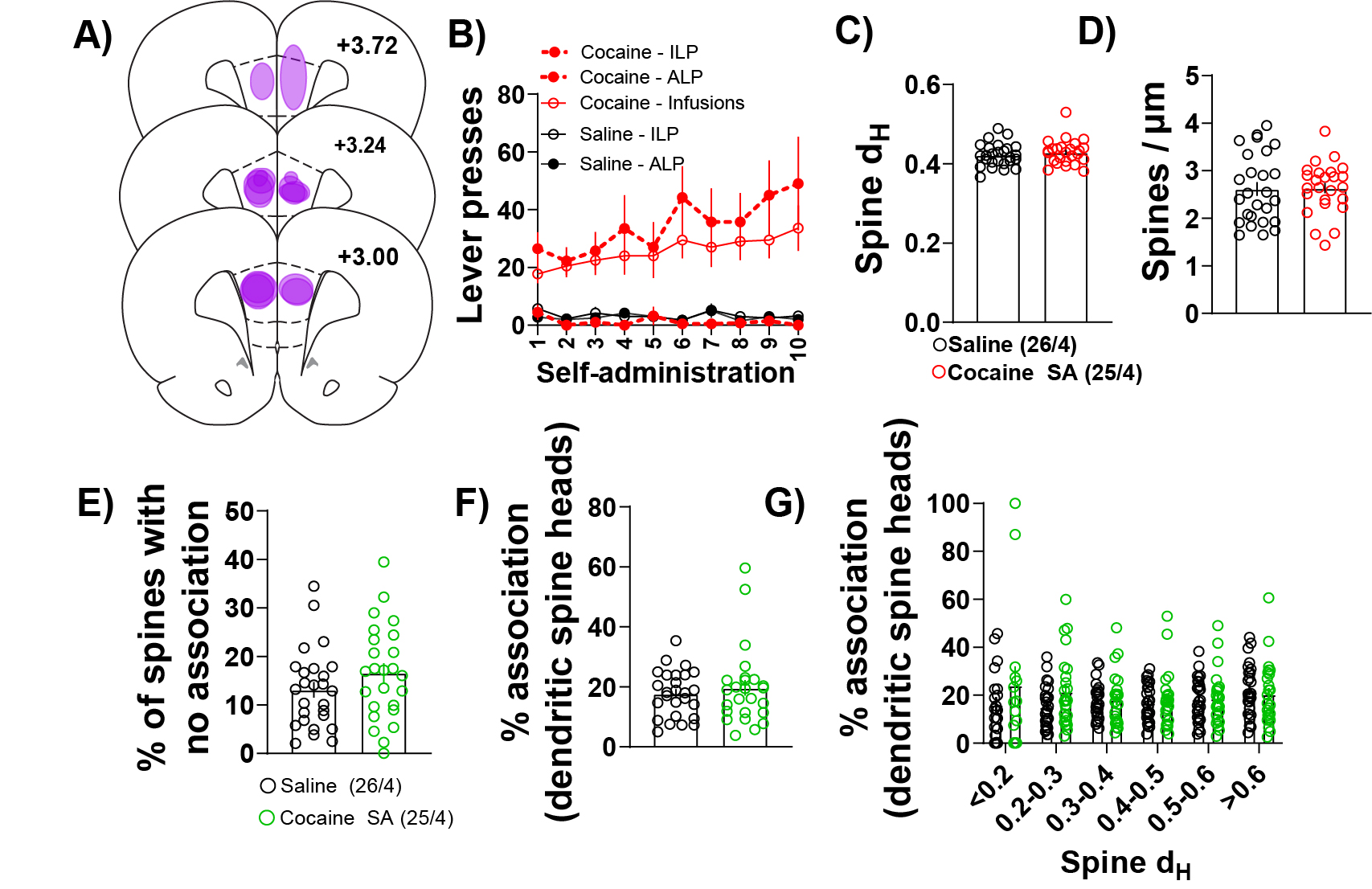

Supplement: Supplementary file 6 [file Image_5.JPEG]
